# Literature-derived criteria and expert-perceived influence structure for age-friendly living environments among older adults living with HIV in China: a text-mining, fuzzy Delphi, and fuzzy DEMATEL study

**DOI:** 10.3389/fpubh.2026.1873646

**Published:** 2026-09-22

**Authors:** Quan Wen, Jing Zhang, Mazran Ismail, Muhammad Hafeez Abdul Nasir, Yanting Wu

**Affiliations:** 1 School of Art and Design, Henan Industry and Trade Vocational College, Zhengzhou, China; 2 School of Housing, Building and Planning, Universiti Sains Malaysia, Penang, Malaysia; 3 Internal Medicine Department, Kaifeng Central Hospital, Kaifeng, China

**Keywords:** age-friendly living environment, China, expert judgement, fuzzy Delphi method, fuzzy DEMATEL, older adults living with HIV, text mining

## Abstract

**Background:**

Older adults living with HIV (OALHIV) may experience the demands of long-term HIV care together with frailty, stigma, privacy concerns, and barriers in their residential environment. This study developed an exploratory set of age-friendly living-environment criteria and examined how experts perceived influence among the resulting domains. It did not test epidemiological causality or measure regional service performance.

**Methods:**

An English-language Web of Science corpus covering 2003–2023 was processed with term co-occurrence mapping and clustering. Fuzzy Delphi method (FDM) ratings from 28 experts were used to screen candidate criteria, followed by domain-level fuzzy decision-making trial and evaluation laboratory (FDEMATEL). Sensitivity analyses examined the FDM cutoff and fuzzy-attitude parameter, and FDEMATEL was calculated using the standard zero-diagonal convention.

**Results:**

The workflow included 1,487 records, 89 cleaned terms, 43 second-round candidates, and 22 retained criteria in five domains. The second-round mean FDM threshold was 0.4729. The same 22 criteria were retained for fixed thresholds from 0.453 to 0.478, for recalculated fuzzy-attitude parameters from 0.40 to 0.60, and under leave-one-criterion-out recalculation. In the zero-diagonal FDEMATEL analysis, healthcare accessibility (*D*−*R* = 0.320), social support network (0.683), and digital inclusion and assistive technology (0.866) occupied net-influencing positions; built-environment adaptation (−1.170) and psychological wellbeing and destigmatization (−0.699) occupied net-influenced positions. The sign classification matched the originally reported calculation, although magnitudes changed.

**Conclusions:**

The framework is hypothesis-generating and reflects literature-derived concepts and expert judgement, not demonstrated causal effects or the directly reported priorities of OALHIV. Patient and community co-design, field measurement, and external validation are required before the criteria can guide resource allocation.

## 1 Introduction

Combination antiretroviral therapy has enabled many people with HIV to live into older age, making multimorbidity, frailty, functional limitation, and continuity of care increasingly relevant to HIV services (1–3). In China, registry and meta-analytic evidence indicates an important and geographically heterogeneous public-health burden among older adults, including spatial clustering and late diagnosis in some settings (4–7).

The ecological theory of aging conceptualizes wellbeing as a function of the fit between personal competence and environmental press (8, 9). The World Health Organization similarly treats functional ability as an interaction between individual capacities and physical, social, and service environments (10, 11). For OALHIV, environmental press may include travel to HIV services, coordination of geriatric and HIV care, inaccessible housing, limited digital access, disclosure risk, stigma, and insufficient social support. These are plausible and interconnected pressures, but their relevance and direction cannot be inferred from theory alone.

Empirical work illustrates the breadth of this problem. Older adults with HIV report geriatric syndromes and functional limitations that can interact with housing and mobility barriers (1, 2). Social support can facilitate engagement in antiretroviral therapy while disclosure can also expose individuals to stigma (12). In China, loneliness and perceived discrimination have been associated with psychosocial and cognitive outcomes, although the cross-sectional designs do not establish causal effects (13, 14). Telehealth can reduce travel burdens, but device access, connectivity, digital skills, and privacy remain limiting conditions for older users (15).

Several studies by the present author group provide adjacent, but distinct, methodological context. These include multidimensional residential-environment assessment (16), natural-language processing for age-friendly facility evaluation (17), SciBERT-assisted public-health framework development (18), and representation learning for community public-health resilience (19). The published AFLE-HIV study developed an operational assessment framework using a different corpus, panel, indicator structure, and field-validation pathway (20). The present study is complementary: it examines literature-derived candidate concepts and an expert-perceived domain-level influence structure. It does not present the AFLE-HIV instrument again, and it does not include field validation.

The regional comparison included in the initial analysis was excluded because author-affiliation publication patterns cannot validly represent local living-environment conditions or service needs. The present study is therefore limited to criterion development and expert-perceived influence.

The revised study therefore addresses two questions:

1. Which literature-derived criteria reached the reported expert-consensus threshold for an age-friendly living-environment framework for OALHIV?
2. What net-influencing and net-influenced positions were assigned to the five domains by the aggregate expert-judgement matrix, and how sensitive was that classification to correction of the matrix diagonal?

The intended contribution is a transparent, exploratory framework that can be tested with OALHIV and community stakeholders. Terms such as “causal model,” “causal determinant,” and “regional priority” are deliberately avoided because the design does not support those claims.

## 2 Conceptual and methodological background

### 2.1 Person–environment fit for older adults living with HIV

Person–environment fit provides a coherent thread connecting the five domains. Healthcare accessibility and digital systems can alter the effort required to obtain care; family, peer, and community networks can buffer social pressure; accessible housing and public space can reduce physical demands; and privacy, counseling, and anti-discrimination mechanisms can influence whether services feel usable. The five domains should therefore be treated as provisional components of environmental fit rather than as independent determinants.

The WHO age-friendly framework covers outdoor spaces and buildings, transportation, housing, social participation, respect and inclusion, civic participation, communication, and community support and health services (10). HIV-related care continuity, privacy, and stigma cut across several of these domains. This cross-cutting character is why a simple checklist may miss perceived interdependence, but it is also why OALHIV must participate directly in later validation.

### 2.2 Evidence for the candidate domains

Healthcare accessibility encompasses antiretroviral therapy, geriatric-HIV coordination, affordability, community service capacity, and follow-up. Comprehensive geriatric assessment has been proposed for aging with HIV because disease-specific care alone may not capture frailty and functional needs (3). Social support includes family, peers, mutual-aid groups, volunteers, and government–non-governmental organization coordination; its effects can be beneficial but are contingent on disclosure safety and stigma (12, 21).

Built-environment adaptation addresses barrier-free access, indoor safety, public space, and residential isolation. Psychological wellbeing and destigmatization include counseling, privacy, screening, and anti-discrimination. The exclusion of stigma and loneliness as stand-alone FDM criteria must not be read as evidence that they are unimportant: both remain conceptually central and are partly represented by privacy, anti-discrimination, peer support, and residential-isolation criteria (13, 14). Digital inclusion includes telemedicine, monitoring, emergency systems, and digital-literacy support; access benefits cannot be assumed where skills, devices, connectivity, or trust are absent (15).

### 2.3 Why text mining, FDM, and FDEMATEL were sequenced

The three methods answer different questions. VOSviewer term mapping identifies recurring concepts and co-occurrence structure in a defined corpus; it does not validate criteria or causal relations (22–24). FDM then formalizes consensus screening under linguistic uncertainty (25, 26). FDEMATEL organizes directional judgements into a driver–effect structure (27, 28). Its outputs describe the structure implied by expert ratings; they are not epidemiological, experimental, or temporal causal estimates.

## 3 Materials and methods

### 3.1 Design and evidential scope

The study was an exploratory framework-development exercise with three stages: (1) corpus-based term identification and clustering; (2) two-round FDM screening; and (3) FDEMATEL analysis of five higher-order domains. Figure 1 distinguishes the source and inferential limit of each stage. No data were collected from OALHIV, and no patient-level, housing, service-performance, or outcome data were analyzed.

**Figure 1:**
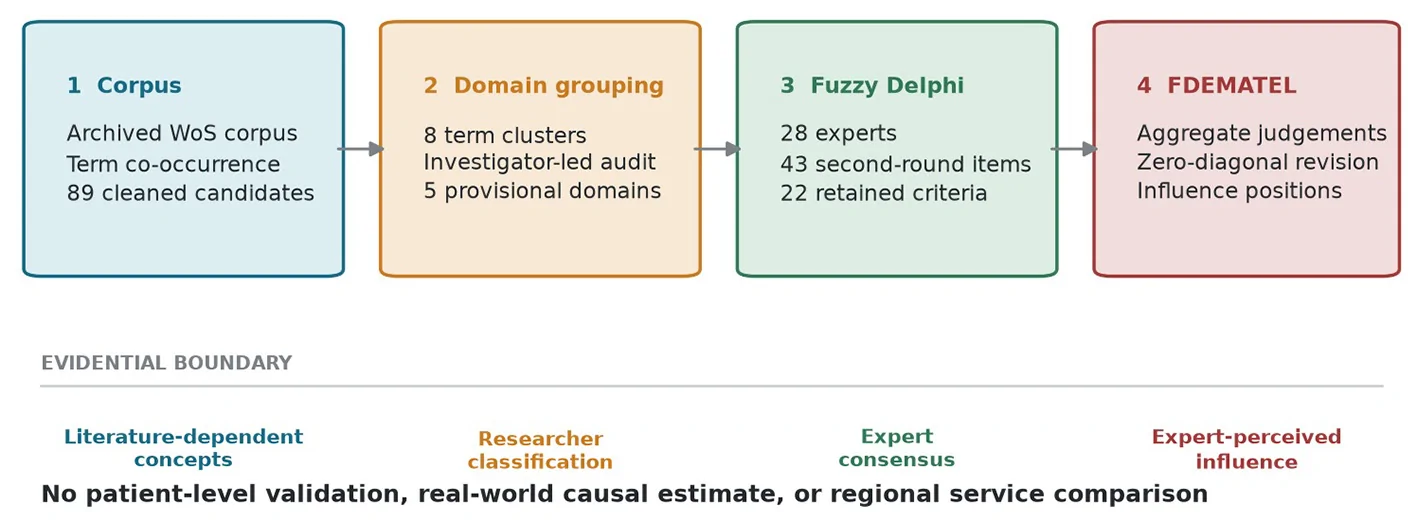
Analytical workflow. Text mining generated corpus-dependent candidate concepts, FDM screened them by expert consensus, and FDEMATEL summarized expert-perceived domain influence. The workflow supports exploratory framework development rather than causal or regional inference.

### 3.2 Literature corpus and term mapping

The study protocol reports a Web of Science Core Collection search conducted on 15 March 2024 for English-language articles and reviews published from 2003 through 2023. The recorded query was:

(“age-friendly” OR “aging in place”) AND (“HIV” OR “AIDS”) AND (“elderly” OR “older adult*”) AND (“living environment” OR “housing” OR “residential” OR “community”).

VOSviewer version 1.6.19 was used for term identification, co-occurrence mapping, and clustering. Study records state that 1,487 records produced 12,860 raw terms; a minimum occurrence threshold of 10 left 195 terms, and manual removal of duplicates, synonyms, generic terms, and out-of-scope terms left 89 candidates. Documentation available for verification records the search protocol, software version, occurrence threshold, and aggregate outputs, but not a complete software-setting log for counting mode, clustering resolution, minimum cluster size, and random seed. The eight-cluster solution is therefore treated as a descriptive output of the original analysis.

### 3.3 Cluster-to-domain consolidation

The 89 terms occurred in eight reported clusters with sizes 17, 15, 16, 16, 9, 7, 6, and 3. Clusters 1–5 corresponded directly to the five substantive domains. Clusters 6–8 contained broad aging, care-system, and health-behavior concepts and were reviewed term by term rather than assigned wholesale. Table 1 records the decision rule and makes its investigator-led nature explicit. The analysis did not include a goodness-of-fit statistic or independent coder-agreement measure; the consolidation should therefore be regarded as a transparent conceptual classification, not a statistically validated factor solution.

**Table 1:** Audit trail from eight reported term clusters to five provisional domains.

Cluster	Terms, *n*	Primary handling	Rationale and limitation
1	17	A1 Healthcare accessibility	ART, geriatric assessment, primary/community care, affordability, follow-up, and coordination formed a service-access group
2	15	A2 Social support network	Family, peer, volunteer, mutual-aid, NGO, and community-organization terms formed a support-network group
3	16	A3 Built-environment adaptation	Barrier-free design, home modification, safety, public space, transport, and isolation concerned physical or residential settings
4	16	A4 Psychological wellbeing and destigmatization	Depression, anxiety, stigma, disclosure, counseling, privacy, and wellbeing concerned psychosocial conditions
5	9	A5 Digital inclusion and assistive technology	Telemedicine, monitoring, digital literacy, mobile health, wearables, and emergency systems concerned technology access and use
6	7	Term-level review for A1/A3 or exclusion	Multimorbidity informed A1; functional capacity, disability, and frailty informed A3; broad aging labels were not treated as criteria merely because they formed a cluster
7	6	Term-level review for A1/A2/A3 or exclusion	Community and long-term care informed service/support domains; age-friendly-city and urban-renewal terms informed A3; framework labels were not retained automatically
8	3	Term-level review, then FDM	Polypharmacy, nutrition, and physical activity were considered as health-related candidates; none was automatically assigned or retained solely on cluster membership

### 3.4 Expert panel and ethics

The aggregate profile included 28 experts with at least 10 years of relevant experience: six in gerontology, five in HIV clinical medicine, five in urban planning, five in social work, and seven in public health. Experience ranged from 10 to 25 years, and the profile recorded university, research-institute, hospital, public-health agency, design-institute, and non-governmental-organization affiliations across Chinese regions (Table 2). The panel was used for both FDM and FDEMATEL.

**Table 2:** Aggregate expert-panel profile reported in the study materials.

Field	*n*	Experience, years	Main affiliation types	Recorded regional coverage
Gerontology	6	10–25	University/research institute	East, Central, West
HIV clinical medicine	5	12–22	Hospital/public-health agency	East, Central, South
Urban planning	5	10–20	University/design institute	East, Central, Northeast
Social work	5	11–18	University/NGO	Central, West, South
Public health	7	10–23	University/public-health agency	Multiple regions

The available aggregate records describe panel eligibility and composition but do not support detailed retrospective reporting of invitation and response flow, participant-level geographic distribution, or participant-level conflict declarations. This documentation boundary is considered when assessing panel representativeness. The Research Ethics Committee of Henan Industry and Trade Vocational College approved the study (HNGM-2022-ETH-018). All participating experts provided written informed consent. The study involved no patients, clinical interventions, or patient records.

### 3.5 Fuzzy Delphi method

Experts rated candidate importance with five linguistic categories. For FDM, very low, low, moderate, high, and very high were represented by triangular fuzzy numbers (0, 0, 0.25), (0, 0.25, 0.5), (0.25, 0.5, 0.75), (0.5, 0.75, 1), and (0.75, 1, 1), respectively. For criterion *i* and expert *h*, the rating was *j*_*ih*_ = (*o*_*ih*_, *p*_*ih*_, *n*_*ih*_). Ratings were aggregated as shown in Equation 1.


$$\begin{array}{l} o_{i}=\underset{h}{\min}\left(o_{ih}\right),\;p_{i}={\left(\overset{l}{\underset{h=1}{\prod }}p_{ih}\right)}^{1/l},\;n_{i}=\underset{h}{\max}\left(n_{ih}\right). \end{array}$$ (1)

With the attitude parameter set at ϵ = 0.5, the lower value, upper value, and convex-combination score were calculated using Equations 2–4.


$$\begin{array}{lll} l_{i} & = & o_{i}+\epsilon \left(p_{i}-o_{i}\right), \end{array}$$ (2)

$$\begin{array}{lll} u_{i} & = & n_{i}-\epsilon \left(n_{i}-p_{i}\right), \end{array}$$ (3)

$$\begin{array}{lll} D_{i} & = & \epsilon \left[u_{i}+\left(1-\epsilon \right)l_{i}\right]. \end{array}$$ (4)

The round-specific threshold was defined as the mean score, as shown in Equation 5.


$$\begin{array}{l} \vartheta =\frac{1}{k}\overset{k}{\underset{i=1}{\sum }}D_{i}, \end{array}$$ (5)

and a candidate was retained when *D*_*i*_≥ϑ. The first-round summary states that 43 of 89 candidates were retained at ϑ_1_ = 0.431. Because the complete aggregate item table is available for the second round, the detailed sensitivity assessment focuses on its 43 candidates, reported in Appendix 1.

Revision sensitivity analyses used only these aggregate second-round values. We (1) varied a fixed cutoff; (2) recomputed all *D*_*i*_ values and the mean threshold for ϵ = 0.40, 0.45, 0.50, 0.55, and 0.60; and (3) recalculated the mean threshold after omitting each criterion in turn. These analyses assess numerical separation around the available aggregate cutoff, not sensitivity to panel composition.

### 3.6 Fuzzy DEMATEL

For FDEMATEL, the five linguistic influence ratings were represented by (0, 0.1, 0.3), (0.1, 0.3, 0.5), (0.3, 0.5, 0.7), (0.5, 0.7, 0.9), and (0.7, 0.9, 1.0). The submitted analysis used the converting-fuzzy-data-into-crisp-scores procedure described by (28). Expert-level crisp values were averaged to form the direct-relation matrix *Z* = [*z*_*ij*_].

Standard DEMATEL fixes self-influence to zero. The available aggregate matrix included non-zero diagonal values; these were set to zero while the off-diagonal values were retained. The normalization, total-relation matrix, and prominence- relation measures were calculated using Equations 6 and 7.


$$\begin{array}{lll} s & =\underset{i}{\max}\underset{j}{\sum }z_{ij},\;N & =\frac{Z}{s}, \end{array}$$ (6)

$$\begin{array}{lll} T & =N{\left(I-N\right)}^{-1},\;D_{i} & =\underset{j}{\sum }t_{ij},\;R_{i}=\underset{j}{\sum }t_{ji}. \end{array}$$ (7)

*D*_*i*_+*R*_*i*_ represents prominence within the expert-assessed system; *D*_*i*_−*R*_*i*_>0 indicates a net-influencing position and *D*_*i*_−*R*_*i*_ < 0 a net-influenced position. These labels describe directional balance in the judgement matrix and do not demonstrate real-world causality. The zero-diagonal results were compared with both the originally reported values and a direct recalculation from the rounded aggregate matrix with its diagonal retained.

All reanalysis calculations and figures were generated in Python 3.14.5 with NumPy 2.5.0 and Matplotlib 3.11.0. The reproducible script is supplied as figures_revised/generate_revision_analysis.py.

## 4 Results

### 4.1 Term clusters and domain consolidation

The reported aggregate pipeline reduced 12,860 raw terms to 195 terms meeting the occurrence threshold and then to 89 manually cleaned candidates. The eight cluster sizes sum to 89 and are internally consistent with the candidate list. Table 1 provides the cluster-to-domain mapping; the cluster output is summarized in tabular form rather than repeated as a separate network visualization.

### 4.2 FDM screening and sensitivity

The 43 second-round scores had a mean of 0.472907, reported as ϑ_2_ = 0.473. Twenty-two criteria met or exceeded this cutoff (Table 3). The lowest accepted score was 0.478 and the highest rejected score was 0.452, leaving a 0.026 separation between the two groups.

**Table 3:** Twenty-two criteria retained in the second FDM round.

Domain	Code	Criterion	*D*_*i*_
A1	C1	ART accessibility	0.555
	C2	Geriatric HIV outpatient coverage	0.554
	C3	Community health service capacity	0.553
	C4	Healthcare affordability	0.556
	C5	Follow-up management convenience	0.478
A2	C6	Family support level	0.555
	C7	Community mutual-aid organizations	0.557
	C8	Volunteer service coverage	0.553
	C9	Government–NGO collaboration mechanism	0.559
	C10	Peer support groups	0.481
A3	C11	Barrier-free facility completeness	0.554
	C12	Indoor safety modification	0.550
	C13	Community public-space adaptation	0.552
	C14	Residential isolation degree	0.556
A4	C15	Psychological counseling availability	0.487
	C16	Community anti-discrimination atmosphere	0.562
	C17	Privacy-protection mechanism	0.555
	C18	Depression/anxiety screening coverage	0.482
A5	C19	Smart health-monitoring device penetration	0.551
	C20	Telemedicine coverage	0.491
	C21	Digital-literacy training	0.553
	C22	Emergency-call-system coverage	0.496

Figures 2, 3 display all accepted and rejected second-round scores. Retention was unchanged for any fixed threshold from 0.453 through 0.478. At cutoffs of 0.450, 0.480, 0.490, and 0.500, the retained counts were 23, 21, 18, and 16, respectively. Recomputing both scores and the mean threshold for ϵ = 0.40 through 0.60 retained the same 22 criteria. Leave-one-criterion-out thresholds ranged from 0.4708 to 0.4751 without changing any remaining classification.

**Figure 2:**
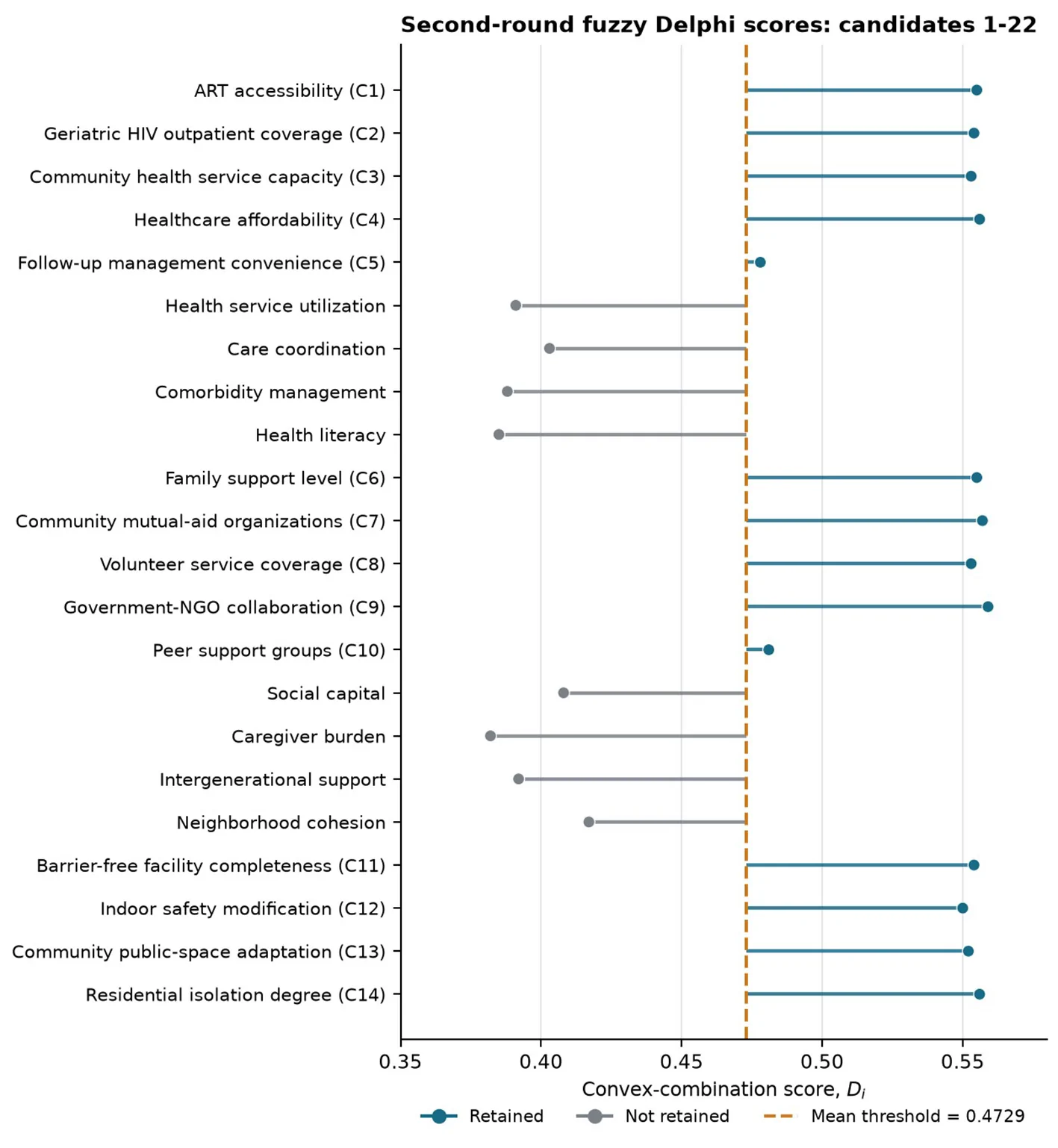
Second-round FDM scores for candidates 1–22 in Appendix 1. The vertical line is the mean threshold (0.4729); blue marks indicate retained criteria and gray marks indicate non-retained candidates.

**Figure 3:**
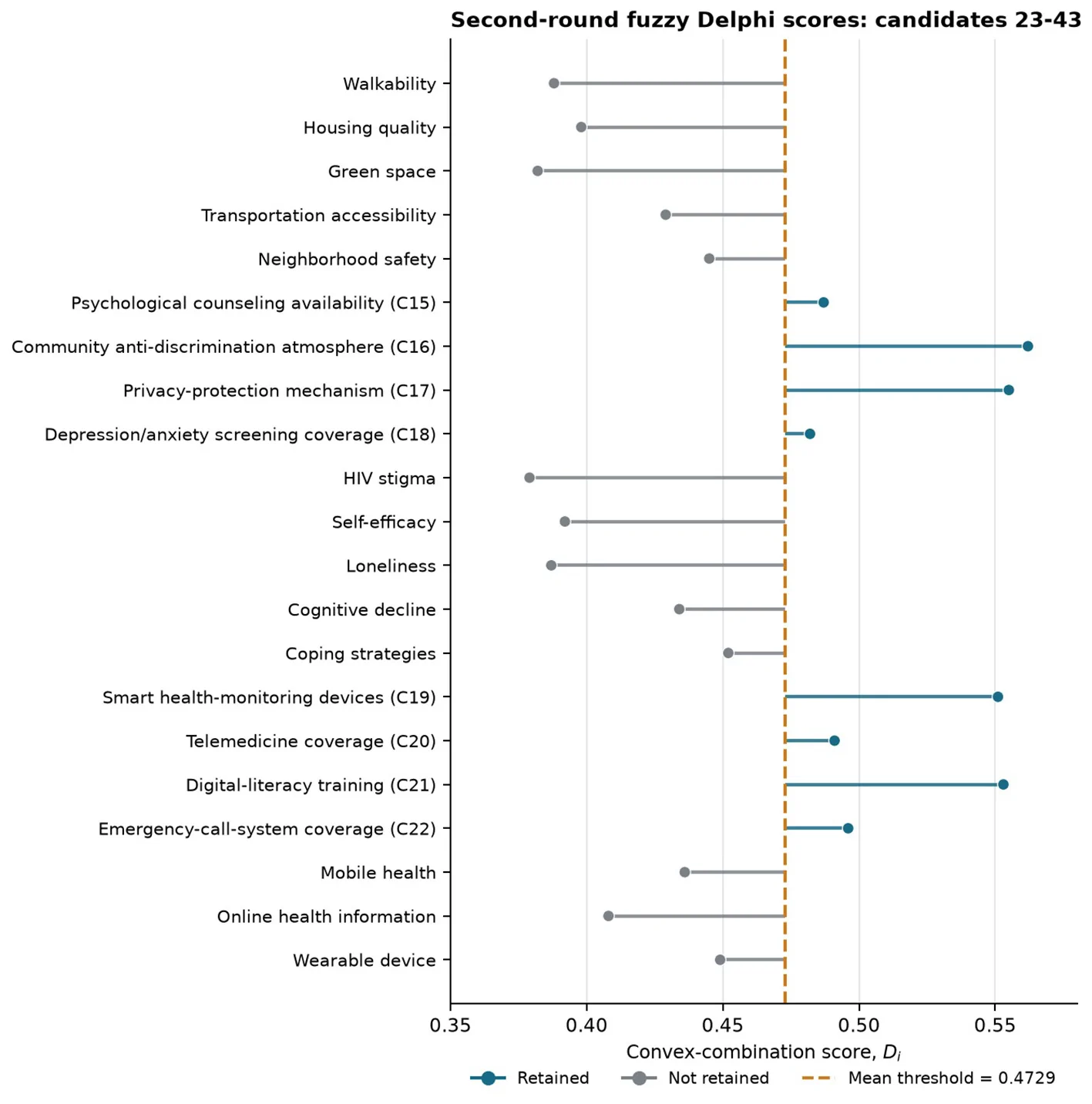
Second-round FDM scores for candidates 23–43 in Appendix 1. The sensitivity analysis uses the complete second-round aggregate table; panel-composition sensitivity was not assessed.

HIV stigma (0.379), loneliness (0.387), self-efficacy (0.392), and cognitive decline (0.434) did not reach the operational consensus threshold as independent criteria. Their exclusion does not imply irrelevance. Stigma and loneliness remain partly represented through anti-discrimination, privacy, peer-support, social-support, and residential-isolation criteria. Self-efficacy and cognitive decline are not directly represented, which narrows content coverage and should be tested in participant-led validation.

### 4.3 Revised FDEMATEL influence structure

Table 4 presents the revised zero-diagonal direct-relation matrix. Its maximum row sum was *s* = 2.489. The resulting total-relation matrix and prominence–relation statistics are shown in Table 5. The prominence-relation pattern is visualized in Figure 4, and the corresponding total-relation matrix is presented as a heatmap in Figure 5.

**Table 4:** Revised aggregate direct-relation matrix with the standard zero diagonal.

Influencing domain	A1	A2	A3	A4	A5
A1	0	0.485	0.671	0.581	0.578
A2	0.631	0	0.631	0.641	0.519
A3	0.428	0.375	0	0.108	0.408
A4	0.395	0.481	0.128	0	0.475
A5	0.636	0.651	0.580	0.622	0

**Table 5:** Revised total-relation matrix and expert-perceived prominence–relation results.

Domain	A1	A2	A3	A4	A5	*D*	*R*	*D + R*	*D-R*	Position
A1 Healthcare accessibility	0.801	0.932	0.993	0.942	0.955	4.623	4.303	8.926	0.320	Net-influencing
A2 Social support network	1.037	0.801	1.013	0.992	0.972	4.815	4.131	8.946	0.683	Net-influencing
A3 Built-environment adaptation	0.672	0.633	0.519	0.546	0.642	3.013	4.183	7.197	−1.170	Net-influenced
A4 Psychological wellbeing/destigmatization	0.725	0.727	0.628	0.564	0.721	3.364	4.063	7.427	−0.699	Net-influenced
A5 Digital inclusion/assistive technology	1.069	1.038	1.030	1.018	0.828	4.983	4.117	9.101	0.866	Net-influencing

**Figure 4:**
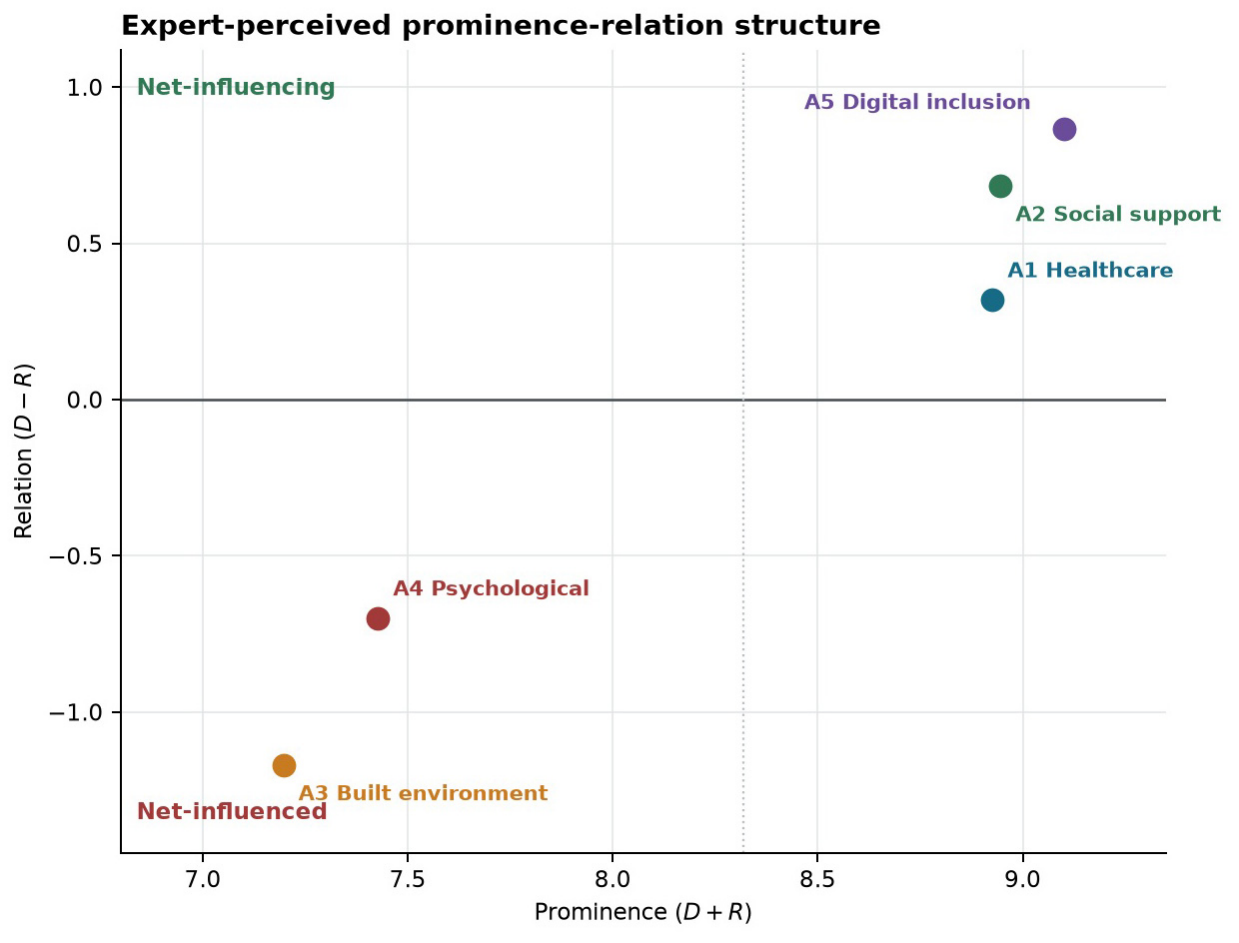
Prominence–relation map from the revised zero-diagonal FDEMATEL calculation. The horizontal coordinate (*D*+*R*) is prominence in the aggregate expert-assessed system; the vertical coordinate (*D*−*R*) indicates net-influencing or net-influenced position. Neither axis is an empirical effect estimate.

**Figure 5:**
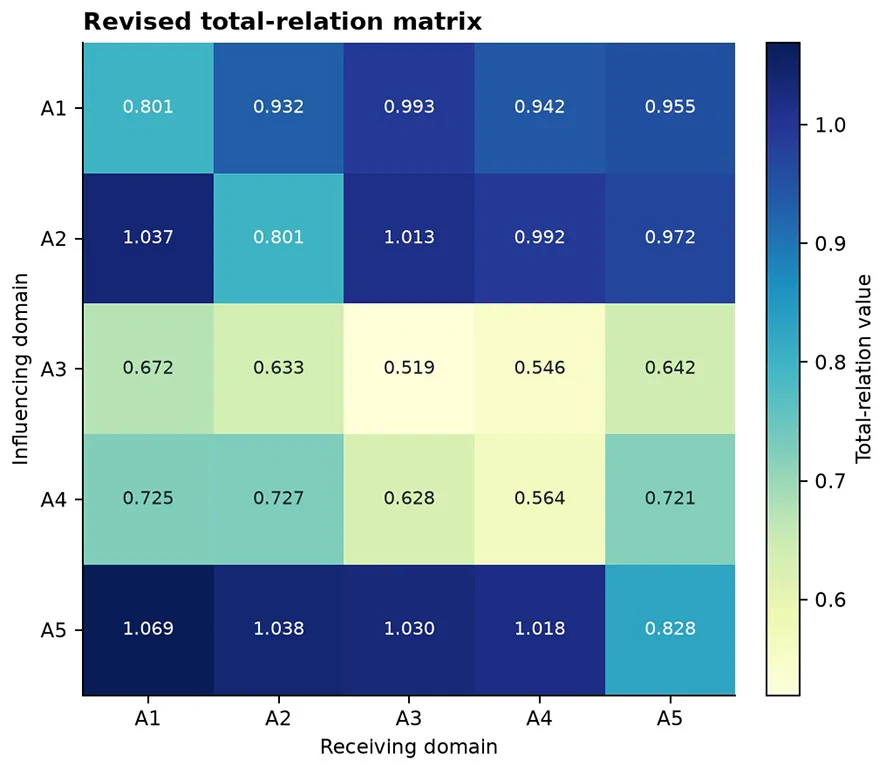
Total-relation matrix after applying the zero-diagonal convention. Cell values are derived from the rounded aggregate direct-relation matrix and should not be interpreted as patient-level associations.

Digital inclusion and assistive technology had the largest positive relation value, followed by social support and healthcare accessibility. Built-environment adaptation had the most negative relation value. These values mean that, within the aggregate expert-judgement system, outgoing assessed influence exceeded incoming assessed influence for A1, A2, and A5, while the reverse held for A3 and A4. They do not show that changing A5 will cause changes in A3 or A4 in practice.

### 4.4 Diagonal-convention sensitivity

The originally submitted table reported *D*−*R* values of 0.435, 0.947, −1.606, −0.954, and 1.178 for A1–A5. Direct recalculation from the rounded reported matrix while retaining its non-zero diagonal produced 0.445, 0.941, −1.608, −0.957, and 1.179. Applying the standard zero-diagonal convention produced 0.320, 0.683, −1.170, −0.699, and 0.866. All three versions assigned A1, A2, and A5 to positive positions and A3 and A4 to negative positions. Thus, the sign classification is robust to this correction. Because the recalculation uses rounded aggregate inputs, interpretation emphasizes this stable classification rather than small differences in magnitude.

## 5 Discussion

### 5.1 What the framework supports

The revised analysis supports a modest conclusion: a panel of 28 experts retained 22 literature-derived criteria, and its aggregate FDEMATEL matrix placed healthcare, social support, and digital inclusion in net-influencing positions relative to built and psychosocial domains. The FDM cutoff was stable within a clearly reported neighborhood. The result can guide hypotheses and the design of field instruments, but it is not evidence of environmental determinants, intervention effects, or causal mechanisms experienced by OALHIV.

The net-influencing position of healthcare accessibility is plausible because continuity of ART and geriatric care can shape whether housing and community supports are usable. The social-support position is also consistent with evidence that support, disclosure, and stigma interact in complex ways (12, 21). Digital inclusion may connect clinical follow-up, peer contact, emergency response, and home-based support, but telehealth evidence also shows that device, connectivity, skill, and privacy barriers can reproduce inequality (15). These external studies make the expert pattern plausible; they do not validate its numerical ordering.

Built-environment adaptation and psychological wellbeing occupied net-influenced positions in the matrix. This should not be translated into lower importance or delayed investment. A net-influenced DEMATEL position reflects balance of judged incoming and outgoing links, not moral priority, effect size, cost-effectiveness, or population need. Barrier-free access, privacy, anti-discrimination, and mental-health support may be necessary irrespective of their matrix position.

### 5.2 Excluded concepts and content validity

Reviewer concern about stigma, loneliness, self-efficacy, and cognitive decline is substantively important. The FDM procedure identifies consensus relative to other candidates; it does not adjudicate whether a concept exists or matters. Chinese evidence documents loneliness and discrimination-related concerns among older people with HIV (13, 14). Future content-validity work should therefore present both retained and excluded concepts to OALHIV and ask whether the current proxy criteria capture their experience. Cognitive impairment and self-efficacy should be reconsidered explicitly because the retained set contains no direct equivalents.

### 5.3 Relationship to the AFLE-HIV framework

The present study and AFLE-HIV (20) address the same population but different analytical purposes. AFLE-HIV develops an operational assessment framework and reports a different evidence base, expert panel, indicator structure, and validation pathway. The current study uses a 1,487-record corpus, FDM screening, and domain-level FDEMATEL to generate an influence hypothesis. Its five domains and 22 criteria should not be presented as an independent replication of AFLE-HIV, nor should methodological overlap be treated as a novelty claim. Instead, this analysis can inform which cross-domain relationships require testing when the operational instrument is used in communities.

### 5.4 Implications for the next empirical study

The next step should be co-design rather than immediate policy ranking. First, OALHIV, caregivers, HIV clinicians, geriatric practitioners, community workers, and housing professionals should review wording, missing concepts, disclosure safety, and respondent burden. Second, a multisite study should measure housing, service access, digital access, social support, stigma, mental health, and patient-prioritized outcomes directly. Third, the five-domain structure should be assessed with content-validity indices, reliability analysis, and an appropriate latent-variable or network model. Fourth, intervention pilots should test specific, ethically acceptable combinations such as telehealth plus digital-skills support, or privacy-protective community care plus peer navigation. No geographic allocation should be inferred from the removed author-affiliation analysis.

### 5.5 Strengths and limitations

The study has three strengths. It separates corpus evidence, expert consensus, and expert-perceived influence; it provides a full second-round FDM table and several threshold checks; and it evaluates the DEMATEL diagonal convention transparently.

Several limitations guide interpretation. First, restriction to Web of Science, English-language publications, and a 2003–2023 window may omit PubMed, Scopus, Chinese-language, and gray literature. The available documentation does not include a complete VOSviewer setting log, so the cluster output is treated as descriptive; manual term cleaning and cluster consolidation may also introduce investigator judgement.

Second, the expert evidence is available at aggregate panel level. Recruitment-flow detail and participant-level ratings were not part of the analytic dataset, limiting assessment of within-panel heterogeneity and sensitivity to a differently composed panel. The disciplinary and regional profile in Table 2 should therefore be considered when judging transferability.

Third, no OALHIV, caregivers, or community residents participated in framework validation. The criteria may therefore miss lived priorities or encode professional assumptions. There is no external validation, patient-level measurement, temporal design, or intervention comparison.

Fourth, the submitted direct-relation matrix used non-zero diagonal entries and the reported total-relation matrix was not exactly reproducible from rounded values. The zero-diagonal correction preserved the sign classification but changed magnitudes. Results should be treated as approximate aggregate judgements.

Finally, the regional component was not retained because author-affiliation publication frequencies cannot identify environmental quality, service need, or policy gaps. The revised manuscript makes no regional comparison.

## 6 Conclusion

This study identifies 22 literature-derived, expert-retained criteria in five domains and reports an exploratory expert-perceived influence structure for age-friendly living environments among OALHIV in China. Threshold checks support the local stability of the second-round FDM split. Correcting the FDEMATEL diagonal preserves the positive positions of healthcare accessibility, social support, and digital inclusion and the negative positions of built-environment adaptation and psychological wellbeing, while changing their magnitudes. These are judgement-structure findings, not causal effects or policy priorities. The framework requires co-design with OALHIV, recovery or prospective collection of auditable source data, and multisite field validation before application.

## Figures and Tables

**Table A1:** All 43 aggregate second-round FDM candidates.

Candidate	*l*_*i*_	*u*_*i*_	*D*_*i*_	Decision
ART accessibility (C1)	0.362	0.929	0.555	Retained
Geriatric HIV outpatient coverage (C2)	0.348	0.934	0.554	Retained
Community health service capacity (C3)	0.355	0.929	0.553	Retained
Healthcare affordability (C4)	0.340	0.942	0.556	Retained
Follow-up management convenience (C5)	0.302	0.805	0.478	Retained
Health service utilization	0.198	0.683	0.391	Not retained
Care coordination	0.215	0.699	0.403	Not retained
Comorbidity management	0.188	0.682	0.388	Not retained
Health literacy	0.172	0.684	0.385	Not retained
Family support level (C6)	0.341	0.940	0.555	Retained
Community mutual-aid organizations (C7)	0.336	0.946	0.557	Retained
Volunteer service coverage (C8)	0.350	0.931	0.553	Retained
Government–NGO collaboration (C9)	0.329	0.954	0.559	Retained
Peer support groups (C10)	0.298	0.813	0.481	Retained
Social capital	0.210	0.711	0.408	Not retained
Caregiver burden	0.183	0.673	0.382	Not retained
Intergenerational support	0.195	0.687	0.392	Not retained
Neighborhood cohesion	0.217	0.726	0.417	Not retained
Barrier-free facility completeness (C11)	0.345	0.936	0.554	Retained
Indoor safety modification (C12)	0.360	0.920	0.550	Retained
Community public-space adaptation (C13)	0.352	0.928	0.552	Retained
Residential isolation degree (C14)	0.338	0.943	0.556	Retained
Walkability	0.189	0.682	0.388	Not retained
Housing quality	0.201	0.696	0.398	Not retained
Green space	0.183	0.673	0.382	Not retained
Transportation accessibility	0.228	0.744	0.429	Not retained
Neighborhood safety	0.241	0.770	0.445	Not retained
Psychological counseling availability (C15)	0.308	0.820	0.487	Retained
Community anti-discrimination atmosphere (C16)	0.321	0.964	0.562	Retained
Privacy-protection mechanism (C17)	0.343	0.939	0.555	Retained
Depression/anxiety screening coverage (C18)	0.305	0.812	0.482	Retained
HIV stigma	0.180	0.668	0.379	Not retained
Self-efficacy	0.195	0.687	0.392	Not retained
Loneliness	0.188	0.680	0.387	Not retained
Cognitive decline	0.232	0.752	0.434	Not retained
Coping strategies	0.248	0.780	0.452	Not retained
Smart health-monitoring device penetration (C19)	0.355	0.925	0.551	Retained
Telemedicine coverage (C20)	0.310	0.827	0.491	Retained
Digital-literacy training (C21)	0.348	0.932	0.553	Retained
Emergency-call-system coverage (C22)	0.315	0.835	0.496	Retained
Mobile health	0.235	0.755	0.436	Not retained
Online health information	0.210	0.711	0.408	Not retained
Wearable device	0.245	0.776	0.449	Not retained

The threshold was 0.4729.

## Data Availability

The original contributions presented in the study are included in the article/supplementary material, further inquiries can be directed to the corresponding authors.
